# Biocontrol Properties of Basidiomycetes: An Overview

**DOI:** 10.3390/jof3010002

**Published:** 2017-01-10

**Authors:** Subramaniyan Sivanandhan, Ameer Khusro, Michael Gabriel Paulraj, Savarimuthu Ignacimuthu, Naif Abdullah AL-Dhabi

**Affiliations:** 1 Entomology Research Institute, Loyola College, Nungambakkam, Chennai 600034, Tamil Nadu, India; sivanandhan2691@gmail.com (S.S.); gabriel_paulraj@yahoo.com (M.G.P.); 2 Department of Plant Biology and Biotechnology, Loyola College, Nungambakkam, Chennai 600034, Tamil Nadu, India; armankhan0301@gmail.com; 3 The International Scientific Partnership Program (ISPP), King Saud University, Riyadh 11451, Saudi Arabia; 4 Department of Botany and Microbiology, College of Science, King Saud University, P. O. BOX 2454, Riyadh 11451, Saudi Arabia; naalharbi@ksu.edu.sa

**Keywords:** biocontrol properties, basidiomycetes, anti-phytofungal activity, anti-phytobacterial activity, anti-phytoviral activity, phytonematicidal activity, mosquito larvicidal activity

## Abstract

In agriculture, there is an urgent need for alternate ecofriendly products to control plant diseases. These alternate products must possess preferable characteristics such as new modes of action, cost effectiveness, biodegradability, and target specificity. In the current scenario, studies on macrofungi have been an area of importance for scientists. Macrofungi grow prolifically and are found in many parts of the world. Basidiomycetes (mushrooms) flourish ubiquitously under warm and humid climates. Basidiomycetes are rich sources of natural antibiotics. The secondary metabolites produced by them possess antimicrobial, antitumor, and antioxidant properties. The present review discusses the potential role of Basidiomycetes as anti-phytofungal, anti-phytobacterial, anti-phytoviral, mosquito larvicidal, and nematicidal agents.

## 1. Introduction

Synthetic chemicals are extensively used in all countries for controlling agricultural pests and plant pathogens [1]. Currently 15% of global crop production is lost due to crop pests [2]. To counteract this, agrochemicals are used in excessive quantities/volumes; it has become apparent that these chemicals are responsible for causing environmental pollution. They leave their residues in food [3]. Unrestrained application of synthetic chemicals causes pesticide resistance, toxicity to humans, plants, and animals, and therefore they are regarded as ecologically unacceptable [4].

Mosquitoes are the most well-known vectors of disease causing pathogens which affect millions of people every year [5]. In India, major diseases are caused by mainly three types of mosquitoes [6], namely *Aedes aegypti* L., *Anopheles stephensi* (Liston), and *Culex quinquefasciatus* (Say). *Ae. aegypti* is a known vector for dengue and chikungunya virus. The malarial parasite is transmitted by *An. stephensi* and the filarial nematode is transmitted by *Cx. quinquefasciatus*. Prevention of mosquito borne diseases is important in order to improve public health, and it is primarily achieved by controlling the vector mosquito population. In recent years, mosquito control programs have encountered failures due to the rapid development of pesticide resistance in mosquitoes [7].

Nematodes have been present for nearly a billion years and are known to cause severe losses to farmers [8]. They feed on many, if not all plants [9]. This pathogen is predominantly part of class Chromodorea, order Rhabdihida [10]. Due to chemical contamination of soil caused by commercial nematicides, newer sources of eco-friendly biomolecules are required in the future.

**Figure 1:**
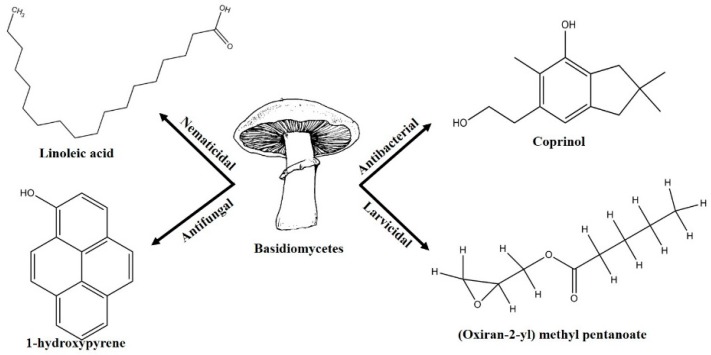
Some of the biocontrol properties of Basidiomycetes compounds.

Pest and pathogen diversities are continuously expanding and new strains are continuously evolving over time [11]. Scientists are looking for safe and more potent alternate products for controlling plant pathogens and pests. The use of natural products for pest control is ideal for sustainable agricultural production with minimum damage to the environment [12]. Basidiomycetes are the fruiting bodies of higher fungi [13]. Several compounds have been isolated from wild Basidiomycetes (Figure 1) which showed growth inhibition of bacteria, virus, and fungi (Table 1) and recorded nematicidal and insecticidal properties [14,15,16,17,18,19,20,21,22,23,24,25,26,27]. In the last few years, Basidiomycetes have received great attention due to their medicinal values, easy availability, and lower side effects and toxicity on non-target organisms [28]. There are nearly 140,000 Basidiomycetes species reported among which about 660 species possess medicinal properties [29]. A number of pharmaceutical substances with potent and unique characteristics have been extracted from Basidiomycetes [30]. Higher Basidiomycetes contain active polysaccharides in their fruiting bodies, cultured mycelia, and cultured broth [31]. The present review focused on anti-phytofungal, anti-phytobacterial, anti-phytoviral, phytonematicidal, and mosquito larvicidal activity of Basidiomycetes.

### Plant Fungal Diseases

Fungi may cause catastrophic plant diseases, because many fungi sporulate prolifically and the spores provide copious inoculums which may infect further plants. The time between infection and the production of further infectious propagules (usually spores) may be only a few days [32]. The spores, if they are wettable, may be spread as high-density inoculums in surface water or in droplets by rain-splash [33]. They may produce phytotoxic compounds [34]. Pathogens may draw nutrients away from the plant by the production or induction of growth regulators [32]. *Colletotrichum gloeosporioides* (anamorph) or *Glomerella cingulata* (teleomorph) causes anthracnoses in many tropical and subtropical crops [35]. Considerable variation occurs in culture and host range, with some strains able to attack many host species whereas others, such as those infecting mango, are confined to a single species [36]. Molecular approaches demonstrated that *C. gloeosporioides* infecting *Stylosanthes* in Australia consisted of two clone populations that did not combine readily in the field which had resulted from two separate introductions into the country [37]. Some strains of *C. gloeosporioides* present a considerable threat to crops growing in countries where there is no fallow period corresponding to the winter of temperate climates [38]. Rice is second only to maize in global production [39]. It is more important since it is the staple food for about half of the world’s populations. It is attacked by the Ascomycete fungus *Pyricularia oryzae* (teleomorph), *Magnaporthe grisea*, causing rice blast, resulting in 10%–30% crop loss every year [40]. More than 700 ha of rice of diverse genotypes with varying levels of resistance in Bhutan were affected in 1995 resulting in losses of 1090 tonnes [40]. Other cereals are also affected by *P. oryzae* or similar species [41]. These include finger millet, *Eleusine coracana*, which, when attacked before grain formation, can suffer complete loss of yield [40]. *P. oryzae* caused yield losses up to 40% in Tanzania [42]. Several fungi produce powerful mycotoxins. For example, the Fumonisin toxins were discovered in the Transkei region of South Africa. They were isolated from cultures of *Gibberella fujikuroi* (anamorph) and *Fusarium moniliforme* grown on maize, and the most active compound was designated as fumonisin B1 (FB1) [43]. FB1 is a sphinganine analogue that, in both plant and animal cells, competitively inhibits sphingolipid biosynthesis causing sphingoid bases to accumulate [44]. High levels of virulence for maize were always associated with strains of the fungus that produce fumonisins [45].

## 2. Biocontrol Properties of Basidiomycetes

### 2.1. Anti-Phytofungal Activity (Table 1)

**Figure 2:**
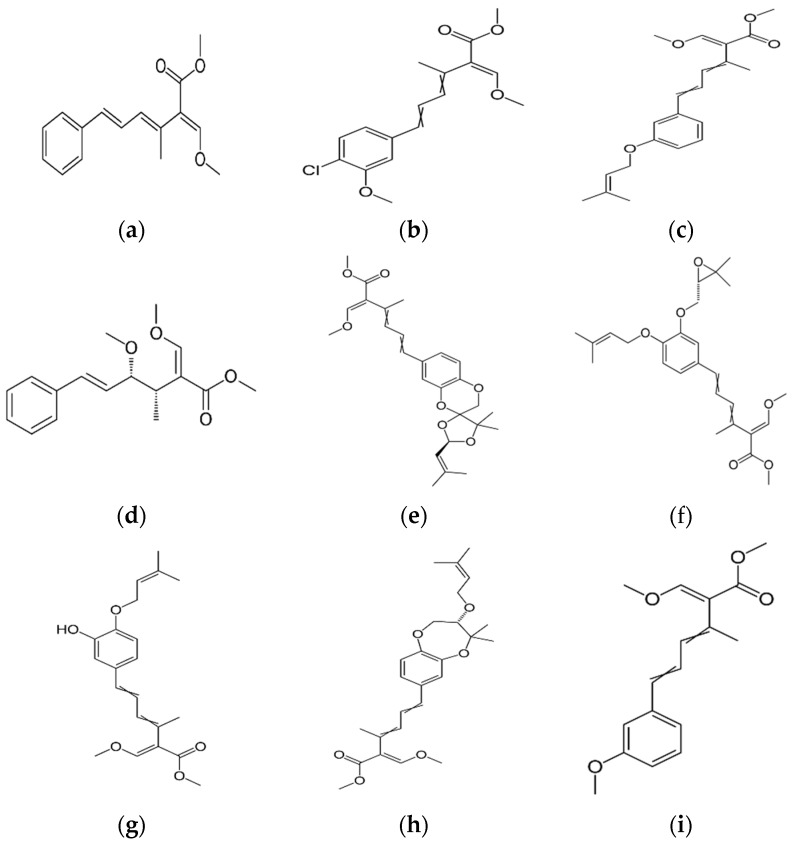
Strobilurin A (**a**); Strobilurin B (**b**); Strobilurin C (**c**); Oudemansin B (**d**); Strobilurin E (**e**); Strobilurin D (**f**); Strobilurin F (**g**); (**h**); Strobilurin H (**i**).

The antifungal agent Grifoline isolated from *Albatrellus dispansus* was effective against several plant fungi in in vitro studies [46]. Phellinsin A was isolated from *Phellinus* sp. It was capable of inhibiting the growth of fungi such as *Gloeosporium orbiculare*, *Pyricularia grisea*, *Thanatephorus cucumeris*, *Aspergillus fumigatus*, and *Trichophyton mentagrophytes* [54]. A major compound in agricultural chemistry was derived from the pine cone fungus, *Strobilurus tenacellus* [55]. Strobilurins are a class of fungicidal compounds, which are extracted from mycelia of the *S. tenacellus*. Strobilurins A (Figure 2a) and B (Figure 2b), that are highly active by inhibiting respiration of yeast and other filamentous fungi [56]. The biochemical activities of strobilurins involve ubihydroquinone cytochrome reductase, which plays a crucial role in respiration [57]. Their activity, however, depends on the presence of (E)-β-methoxyacrylate moiety [58]. Strobilurin fungicides have become valuable tools for managing plant diseases [59]. These strobilurins are site specific (inhibition of mitochondrial respiration) and translaminar (systemic) compounds that provide control of *Oomycota, Ascomycota, Basidiomycota*, and *Deuteromycota* fungi. (E)-β-methoxyacrylates of strobilurin C (Figure 2c) and Oudemansin B (Figure 2d) from cultures of *Xerula pudens* inhibit many phytopathogenic fungi. Like the strobilurins A and B, they have also been shown to inhibit fungal respiration [60]. Strobilurin E (Figure 2e) is another antifungal compound of the (E)-β-methoxyacrylate class extracted from mycelial cultures of *Crepidotus fulvotomentosus*. In addition to inhibiting fungal respiration, it has been shown to induce cell deformations [57]. Strobilurins D (Figure 2f) and F (Figure 2g) are other strobilurins, extracted from mycelial cultures of the Basidiomycete *Merismodes anomala*; they have cytostatic and antifungal antibiotics of the (E)-β-methoxyacrylate class. These strobilurins inhibit many fungi, and like strobilurins A and B, they also are potent inhibitors of respiration [57,61]. Strobilurin M which was isolated from the *Mycena* sp. showed antifungal and cytostatic activities [61]. Other strobilurins F (Figure 2g), G (Figure 2h), and H (Figure 2i) extracted from culture fluids of *Bolinea lutea* inhibited *Aspergillus fumigatus*, *Botrytis cinerea*, *Microsporum canis*, and *Sporothrix schenckii*. These compounds reduced fungal respiration. However, they might be different from the analogs previously described [58]. Wang et al. reported that 15 kDa antifungal protein designated as Ganodermin, was isolated from *Ganoderma lucidum*. It inhibited mycelial growth of *Botrytis cinerea*, *Fusarium oxyporum*, and *Botryosphaeria berengeriana*. The IC_50_ (concentration which inhibits 50% growth) values of the ganodermin against *B. cinerea*, *Fusarium oxysporum*, and *B. berengeriana* were 15.2–0.7 µM, 12.4–0.3 µM, and 18.1–0.5 µM, respectively. Pleurostrin, an antifungal peptide with about half the size of ganodermin, has been isolated from the oyster Basidiomycetes *Pleurotus ostreatus*. Ganodermin inhibited mycelial growth in the phytopathogenic fungi *Botrytis cinerea*, *F. oxyporum*, and *Peyronellaea arachidicolla*. Very few bioactive proteins, such as a lectin and a ribonuclease, have been isolated from *G. lucidum* [19]. The antifungal activity of culture filtrates, methanol, and water extracts of *Stereum ostrea*, an inedible Basidiomycetes, was tested against three plant fungal pathogens namely *Botrytis cinerea, Colletotrichum miyabeanus*, and *Colletotrichum gloeosporioides* [61].

### 2.2. Anti-Phytobacterial Activities

**Figure 3:**
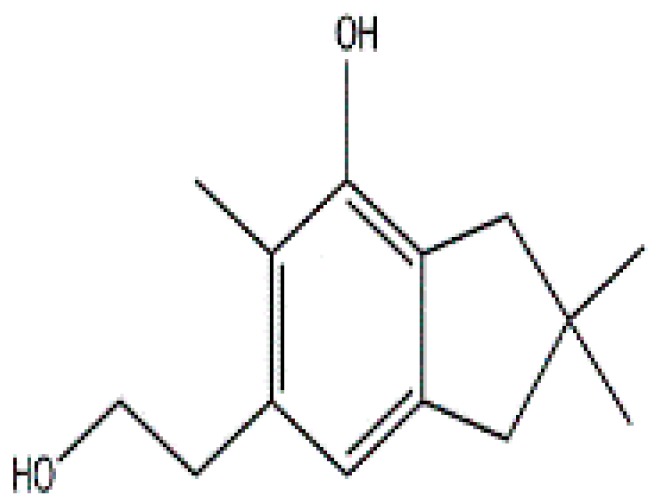
Coprinol.

Secondary metabolites isolated from various Basidiomycetes have been known to show antibacterial properties [62,63,64,65]. Basidiomycetes provide effective and low-cost products for human and plant disease control. Members of Ganodermatales, Poriales, Agaricales, and Stereales show potential antibacterial activity and these may become substitutes for developing new antibiotics [66]. The effect of secondary metabolites of Basidiomycetes has been investigated mainly on human and animal pathogens. Erjave et al. reported that 15 Basidiomycetes extracts showed moderate to high antibacterial activities and three extracts regressed the disease as well as reduced the severity in vitro and in vivo against bacterial wilt disease caused by *Ralstonia solanacearum* [67]. Extracts from *Clytocybe geotropa* showed broad range of inhibition against *R. solanacearum*, *Erwinia carotovora* subsp. *carotovora*, *P. syringae* pv. *syringae*, *X. campestris* pv. *Vesicatoria*, and *Clavibacter michiganensis* subsp. *sepedonicus*. Purified protein, Clitocypin, from *C. geotropa* showed effective inhibition against *C. michiganensis* subsp. *Sepedonicus* [68]. The fungicide strobilurin F 500 enhanced resistance of tobacco to the wild fire pathogen *Pseudomonas syringae* pv. *tabaci* [69]. Coprinol (Figure 3), isolated from *Coprinus* sp., showed inhibitory activity against most of the plant pathogens [70].

### 2.3. Anti-Phytoviral Activity

**Figure 4:**
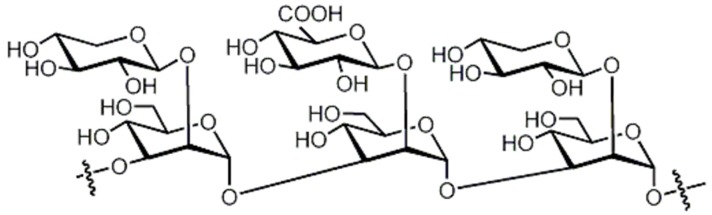
Polysaccharide glucuronoxylomannan (GXM).

Brandt and Piraino (2000) divided the antiviral compounds from fungi into two major classes: (i) biological response modifiers and (ii) viral inhibitors [71]. The effect of Basidiomycete polysaccharides has been investigated using human and animal virus models [72]. *Ganoderma lucidum* and *G. applanatum* strains were able to inhibit the tobacco mosaic virus (TMV) at a concentration of 1000 µg/mL [73]. The filtrate from cultured biomass of the polypore *Fomes fomentarius* was effective against the mechanical transmission of TMV [74]. A new lectin, named AAL (*Agrocybe aegerita* lectin), had been purified from the fruiting bodies of the edible Basidiomycetes *A. aegerita* [26]. Aqueous extracts from *Agaricus brasiliensis* and *Lentinula edodes* fruiting bodies showed antiviral activity against the aphid-borne mosaic virus [75]. The neutral and acid polysaccharides have different characteristics of anti-phytoviral activity. Glucuronoxylomannan (GXM) was considerably less active; in this case, the total preparation occupied an intermediate position, revealing evidence that activity of the total preparation relative to the infectivity of TMV was induced to a greater extent [76]. The effect of Basidiomycete metabolites on plant pathogenic viruses has been poorly studied [75]. A pink quinone, tentatively identified as β-l-glutaminyl-3,4-benzoquinone, present in sporophores of *Agaricus bisporus* is a potent inhibitor of plant virus infections [77]. It showed inhibitory activity to infection of TMV on *Nicotiana glutinosa*. In particular, the acid polysaccharide of GXM (Figure 4) produced by *Tremella mesenterica* consisted of a linear backbone of β-(1→2) (1→4)-linked oligosaccharides of xylose and glucuronic acid [78,79,80].

### 2.4. Phytonematicidal Activity

Researchers throughout the world have become increasingly interested in diminishing the use of chemical strategies against parasites [81,82,83]. A recent study has demonstrated that some species of edible Basidiomycetes such as *Pleurotus* species possess nematicidal activity through the production of a nematode-toxin, which is able to inhibit the nematode movement, allowing hyphal penetration, and finally digesting the body by enzymatic action [84]. Such biological activity could be the result of a self-defense mechanism in Basidiomycetes which acts against the attack of myceliophagous nematodes [85]. Other studies have shown that *P. ostreatus* produces a nematode-toxin similar to peroxides which inhibits the movement of nematodes and subsequently degrades them, reaching a mortality of 95% in the free-living nematode *Panagrellus redivivus* (adults) and the phytopathogenic nematode *Bursaphelenchus xylophilus* [86]. Another study conducted by Palizi et al. showed that *P. eryngii* caused 50% mortality against the phytopathogenic nematode *Heterodera schantii* which caused wilting in sugarcane and other crops. In general, nematicidal activity shown by the different strains of *P. eryngii* ranged between 4.8%–99.6%. The nematicidal effect of specific edible Basidiomycetes could be influenced by a number of factors like temperature, incubation time of the confrontation, inner genetic characteristics of each of the strains, and differences between nematode species used [87]. Some dead larvae observed with mycelium inside their bodies suggested that the larval death was a consequence of body rupture and invasion by fungal mycelia [87]. Mamiy reported that a strain of *Coprinus comatus* immobilized, killed, and consumed the free-living nematode *Panagrellus redivivus* and the root-knot nematode *Meloidogyne arenaria* [88]. The author reported mechanical damage in the free-living nematode *P. redivivus*, 8 h post-confrontation with *C. comatus* mycelia, resulting in 90% nematode immobilization and subsequent degradation, at 24 °C incubation. The strains of the edible *Pleurotus ostreatus* ECS-1123 and ECS-0152, *P. eryngii* ECS-1290 and ECS -1291, *P. cornucopiae* ECS-1328 and ECS-1330, and *Lentinula edodes* ECS-0401 displayed high nematicidal activity with a range of 82% to 99% mortality [84]. Bua-art et al. reported the potential use of bioactive compounds from luminescent Basidiomycete (*Neonothopanus nambi*) for control of plant parasitic root-knot nematode *Meloidogyne incognita*. The results revealed that concentrations of 500 mg/L were highly toxic to *M. incognita* causing 100% mortality within 30 min [89]. 

**Figure 5:**
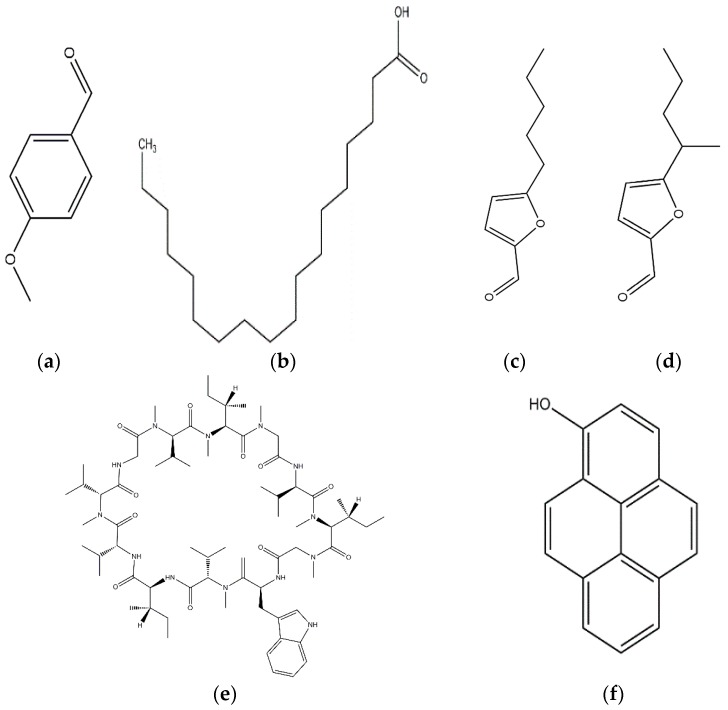
p-anisaldehyde (**a**); Linoleic acid (**b**); 5-pentyl-2-furaldehyde (**c**); 5(4-penteny)-2-furaldehyde (**d**); Omphalotin (**e**); 1-Hydroxypyrene (**f**).

Anisaldehyde, 3-chloro-anisaldehyde, and (4-methoxyphenyl)-1,2-propandiol were isolated from several common wood and forest-litter degrading fungi (e.g., *Pleurotus pulmonarius*, *Bjerkandera adusta*, *Hypholoma fasciculare*, and *Pholiota squarrosa*) [90]. Weak antifungal and nematicidal properties have been described for p-anisaldehyde (Figure 5a) and (4-methoxyphenyl)-1,2-propandiol. Fatty acids like *S*-coriolic acid or linoleic acid (Figure 5b) isolated from *P. pulmonarius* exhibited nematicidal effects against the saprophytic nematode *Caenorhabditis elegans*, with LD_50_ (Median lethal dosage) values of 10 and 5 µg/mL, respectively [91]. These nematicidal effects depended on the degree of unsaturation and the length of the fatty acid. A nematicidal monoterpene, 1,2-dihydroxymintlactone, was isolated from *Cheimonophyllum candidissimum*. It recorded LD_50_ value of 25 µg/mL against *C. elegans* and herbicidal effects against *Setaria italica* and *Lepidium sativum* at concentrations starting from 50 µg/mL [92]. Cheimonophyllons and cheimonophyllal were isolated from the wood-inhabiting Basidiomycete *Cheimonophyllum candidissimum* and they exhibited nematicidal activities against nematode *C. elegans* [92]. The Furaldehydes, 5-pentyl-2-furaldehyde (Figure 5c) and 5(4-penteny)-2-furaldehyde (Figure 5d) isolated from *Irpex lacteus*, exhibited nematicidal activity against *Aphelencoides besseyi* [93]. A nematicidal cyclic peptide omphalotin (Figure 5e) was isolated from biomass after fermentation of *Omphalotus olearius* [94]. 1-Hydroxypyrene (Figure 5f) derived from *Crinipellis stipitaria* showed very strong nematicidal activity against *C. elegans* [53]. The cultural filtrates from *Amauroderma macer*, *Laccaria tortilis*, and *Tylopilus striatulus* showed high nematicidal activity against the pine wood nematode *Bursaphelenchus xylophilus* [95].

### 2.5. Mosquito Larvicidal Activity

**Figure 6:**
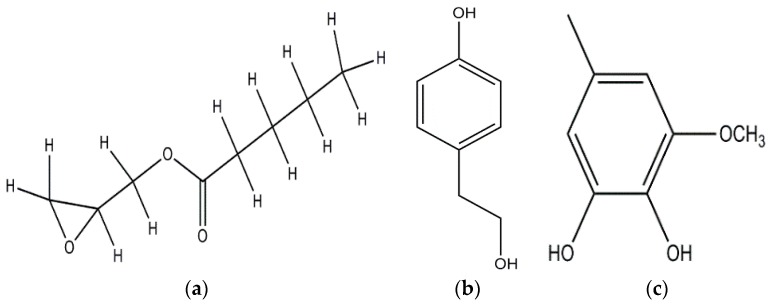
(Oxiran-2-yl) methylpentanoate (**a**); 4-(2-hydroxyethyl) phenol (**b**); 3-methoxy-5-methyl-1,2-benzenediol (**c**).

Few studies have been done to find out the mosquito larvicidal activity of Basidiomycetes extracts. The (Oxiran-2-yl) methylpentanoate (Figure 6a) isolated from submerged culture of *Cyptotrama asprata* showed 100% larvicidal activity at 1.25 ppm (parts per million) concentration against *Aedes aegypti* after 8 h [96]. High larvicidal activities against *Ae. aegypti* and *Anopheles nuneztovari* were observed using the crude extracts of *Pycnoporus sanguineus* and *Pestalotiopsis virgulata* [97]. Crude ethanolic extract of *Lactarius gymnocarpoides* showed maximum larvicidal activity against *Ae. aegypti* [98]. Ethanol extracts of *Amanita phalloides*, *Russula cellulata*, *Lactarius gymnocarpoides*, and *L. densifolius* exhibited weak activity against *Cx. quinquefasciatus* and *Ae. aegypti* [99]. The larvicidal activity of methanolic extract of *G. lucidum* against fourth instar larvae of *Cx. pipiens* was tested post 24 h exposure and it ranged from 18.25% at 0.5 mg·L^−1^ concentration to 100% at 5 mg·L^−1^ concentration [100]. 4-(2-hydroxyethyl) phenol (Figure 6b) and 3-methoxy-5-methyl-1,2-benzenediol (Figure 6c) with LC_50_ values of 231 and 237 ppm, respectively, were isolated from Basidiomycete JO5289, and were found to be active against *Ae. aegypti* larvae after 24 h [101]. *Thaeogyroporus porentosus*, *Xylaria nigripes*, *Chlorophyllum* sp., and *Steccherinum* species had good larvicidal activities against *Ae. aegypti* with mortality ranging from 10%–70% and 18%–90% for 24 and 48 h exposure times, respectively [102].

## 3. Conclusions

Basidiomycetes occupy a prominent position in medical and biological research fields because of their antimicrobial, insecticidal, and nematicidal properties. They are easily available or cultivable and economic. As such, their secondary metabolites with active principles can be produced cost-effectively. The present review clearly shows the various biological properties of Basidiomycetes compounds against plant pathogenic microbes, vector mosquitoes, and nematodes. Discovery of more novel natural products from Basidiomycetes for the control of plant diseases, vector mosquitoes, and nematodes will lead to ecofriendly crop protection methods. The interactions between the bioactive metabolites of Basidiomycetes and host cells should be studied to understand the mechanisms behind their antibacterial, larvicidal, nematicidal, and other activities. The potential bioactive metabolites obtained from Basidiomycetes should be urgently commercialized to reduce the unwanted effects of synthetic chemicals.

## Figures and Tables

**Table 1:** Active compounds isolated from different mushroom species.

Active Compound	Compound Structure	Target Pathogen	Reference
Ganodermin (*Ganoderma lucidum*) Antifungal protein	NA	*Physalospora piricola; Botytis cinerea*	[19]
Pleurostrin (*Pleurotus ostreatus*) Antifungal protein	NA	*Botryosphaeria berengeriana*	[20]
Eryngin (*Pleurotus eryngii*) Antifungal protein	NA	*Mycosphaerella arachidicola*	[20,23]
Antifungal protein mLAP (*Lyophyllum shimeji*)	NA	*Physalospora piricola*	[22]
Lyophyllin (*Lyophyllus shimeji*)	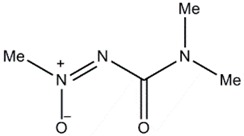	*Physalospora piricola*	[22]
Grifoline (*Albatrellus dispansus*)	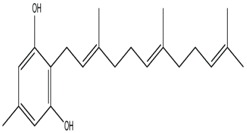	*Alternaria alternate*; *Pyricularia oryzae*; *Rhizoctonia solani*; *Sclerotina sclerotiorum*; *Fusarium graminearum; Botytis cinerea; Gaeumannomyces graminis*; *Gloesporium fructigenum*	[46]
Hypsin (*Hypsizigus marmoreus*) Antifungal protein	NA	*Botryosphaeria berengeriana*; *Botytis cinerea*; *Mycosphaerella arachidicola*	[47]
Rufuslactone (*Lactarius rufus*)	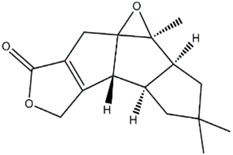	*Alternaria alternata*; *Fusarium graminearum*; *Botytis cinerea; Alternaria brassicae*	[48]
Cordymin (*Cordyceps militaris*) Antifungal protein	NA	*Rhizoctonia solani*; *Mycosphaerella arachidicola*; *Bipolaris maydis*	[49]
p-Hydroxybenzoic and Cinnamic acids (*Ganoderma lucidum*)	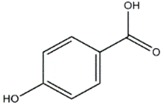	*Trichoderma viride*; *Penicillium ochrochloron*; *P. funiculosum*	[50]
	p-Hydroxybenzoic		
	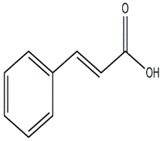		
	Cinnamic acids		
Chrysotrione A and B (*Hygrophorus chrysodon*)	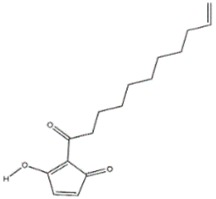	*Fusarium verticillioides*	[50]
	Chrysotrione A		
	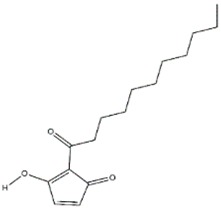		
	Chrysotrione B		
Agrocybin (*Agrocybe cylindracea*)	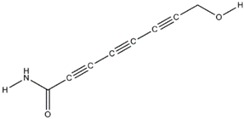	*Mycosphaerella arachidicola*	[51]
Lentin (*Lentinus edodes*) Antifungal protein	NA	*Mycosphaerella arachidicola*	[52]
1-hydroxypyrene (*Cordyceps militaris*)	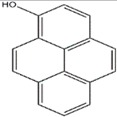	*Fusarium oxysporum*	[53]
Phellinsin A (*Phellinus* sp.)	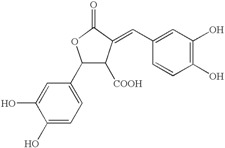	*Gloeosporium orbiculare*; *Pyricularia grisea*	[54]

Note: NA—not available.
